# Cortisol and periodontitis: Prospective observational and Mendelian randomization studies

**DOI:** 10.3389/fendo.2023.1100985

**Published:** 2023-03-15

**Authors:** Sebastian-Edgar Baumeister, Stefan Lars Reckelkamm, Hans-Jörgen Grabe, Matthias Nauck, Johanna Klinger-König, Henry Völzke, Thomas Kocher, Nele Friedrich, Birte Holtfreter

**Affiliations:** ^1^ Institute of Health Services Research in Dentistry, University of Münster, Münster, Germany; ^2^ Department of Psychiatry and Psychotherapy, University Medicine Greifswald, Greifswald, Germany; ^3^ Institute of Clinical Chemistry and Laboratory Medicine, University Medicine Greifswald, Greifswald, Germany; ^4^ DZHK (German Centre for Cardiovascular Research), Partner Site Greifswald, University Medicine, Greifswald, Germany; ^5^ Institute for Community Medicine, University Medicine Greifswald, Greifswald, Germany; ^6^ Department of Restorative Dentistry, Periodontology, Endodontology, and Preventive and Pediatric Dentistry, University Medicine Greifswald, Greifswald, Germany

**Keywords:** cortisol, periodontitis, observational study, mendelian randomization, hypothalamus-pituitary-adrenal (HPA) axis

## Abstract

**Purpose:**

Cortisol has obesogenic, hyperglycemic and immunomodulating effects. Preclinical and observational research suggested that it is associated with periodontitis but the evidence for potential causality in humans is sparse. We triangulated results from prospective observational and Mendelian randomization (MR) analyses to further explore this.

**Methods:**

Using pooled data from 3,388 participants of two population cohort studies embedded in the Study of Health in Pomerania (SHIP) project, we associated serum cortisol levels with periodontal outcomes measured after a median follow-up time of 6.9 years, adjusting for confounding and selection bias using propensity score weighting and multiple imputation. We further examined the effect of genetically proxied plasma morning cortisol levels on periodontitis using two-sample MR of 17,353 cases and 28,210 controls.

**Results:**

In SHIP, we found that cortisol levels were positively associated with follow-up levels of mean clinical attachment level (CAL), deep interdental CAL and bleeding on probing but were unrelated to mean probing pocket depth and deep periodontal pockets. In MR analysis, cortisol was not associated with periodontitis.

**Conclusion:**

The observational study revealed a prospective association of spot cortisol with makers of periodontitis. Contrary to observational studies, genetically instrumented, long-term cortisol was unrelated to periodontitis. Our results find no univocal evidence that cortisol plays a role in periodontitis pathology, casting doubt on cortisol-related pathways.

## Introduction

Cross-sectional observational human and animal research suggested an effect of cortisol on periodontal disease, which is assumed to be mediated by obesogenic, hyperglycemic, and immunomodulating pathways (1–3). Glucocorticoid receptor resistance, a condition of reduced sensitivity of immune cells to glucocorticoids due to chronic cortisol secretion, could additionally contribute to adverse effects on the periodontium (4). Oral tissues have glucocorticoid receptors that respond to the chronic release of glucocorticoids (1). In the oral cavity, glucocorticoids depress immunity by inhibiting the production of secretory immunoglobulins, and neutrophil function, which may impair defense against periodontal microorganisms. Recently, direct response of oral microorganisms to cortisol has been observed (5). Functional metatranscriptomic analysis found that members of the phylum Fusobacterium are activated by cortisol (6). Exposure to cortisol changed the activity profile of the oral microbiome and overactivated the local host immune response, which predisposes to periodontitis (5, 7). Furthermore, glucocorticoid secretion generates reactive oxidative stress *via* increased mitochondrial activity, potentially damaging telomeres and inhibiting telomerase activity, both of which contribute to cellular senescence (8). The lack of diversity in the immune response to infections in aging people with periodontitis may be explained by age-associated immune cell loss selecting lymphocytes with normal telomere length (9).

It is unclear how the findings from the aforementioned *in vivo* and *in vitro* research translates to human populations. Molecular, cellular, and pathophysiological differences between animal models and humans impede the extrapolation of preclinical findings to humans. Furthermore, although previous observational studies suggested an association of circulating and salivary cortisol with periodontitis (10, 11), the available observational studies were of cross-sectional or case-control designs, small samples sizes, and made little effort to adjust for confounding and reverse causation. The current study presents findings of prospective observational and Mendelian randomization (MR) analyses to further strengthen the evidence for cortisol in the etiology of periodontitis in humans.

## Materials and methods

### Observational study

#### Study design, inclusion, and study variables

The Study of Health in Pomerania (SHIP) project comprises two prospective, population-based cohort studies that recruited individuals living in West-Pomerania, Germany. The first sample (SHIP-START-0: 1997-2001; N = 4,308) was drawn from population registries. Study participants were reexamined at regular follow-ups (SHIP-START-1: 2002-2006, N = 3,300; SHIP-START-2: 2008-2012, N = 2,333; SHIP-START-3: 2014–2016, N = 1,178). A second, independent sample (SHIP-TREND-0: 2008-2012; N = 4,420) was recruited from the same study region and reexamined after seven years (SHIP-TREND-1: 2016-2019, N = 2,507). The study has been described in detail previously (12). We combined SHIP-START-2 and SHIP-TREND-0 and performed prospective analysis in the pooled data with follow-up periodontitis outcomes measured after a median follow-up of 6.9 years (25. percentile = 5.2; 75. percentile = 7.3). For the primary complete case analysis, we excluded participants with systemic corticosteroid use (Anatomical Therapeutic Chemical classification code H02) and missing data on periodontal outcomes at follow-up, baseline cortisol or baseline covariates, leaving 3,388 participants (**Table 1**). Participants gave written informed consent, and the Ethics Committee of the University of Greifswald approved the study.

**Table 1:** Baseline descriptive statistics of the Study of Health in Pomerania (SHIP).

	Total	SHIP-Start-0	SHIP-Trend-0
Sample (complete case analysis), n	3388	1307	2081
Mean pocket probing depth, mm	2.5 (0.6)	2.6 (0.5)	2.5 (0.6)
Proportion of sites with pocket probing depth ≥4 mm	0.12 (0.16)	0.12 (0.14)	0.12 (0.17)
Proportion of sites with pocket probing depth ≥6 mm	0.02 (0.06)	0.02 (0.06)	0.02 (0.06)
Mean clinical attachment level, mm	2.4 (1.5)	2.7 (1.4)	2.2 (1.5)
Proportion of interdental clinical attachment level >2mm	0.43 (0.37)	0.52 (0.37)	0.38 (0.36)
Proportion of sites bleeding on probing	0.24 (0.23)	0.25 (0.23)	0.23 (0.23)
Serum cortisol, nmol/l	323.9 (130.1)	307.4 (122.3)	334.1 (133.3)
Age, years	50.8 (13.3)	54.6 (12.1)	48.7 (13.5)
Female sex	1764 (52.1)	668 (53.1)	1082 (51.4)
Smoking status			
Former smoker	1361 (40.2)	536 (42.6)	811 (38.5)
Never smoker	719 (21.2)	224 (17.8)	810 (38.5)
Current smoker	1308 (38.6)	498 (39.6)	483 (23.0)
Alcohol consumption, grams per day	9.5 (13.3)	10.3 (13.6)	8.9 (12.9)
Waist circumference, cm	89. 6 (13.4)	90. 7 (13.5)	88.9 (13.3)
Fasting time, h:min	8:14 (6:14)	4:11 (6:18)	9:53 (5:19)
Time of blood sampling, h:min	9:18 (0:57)	9:04 (1:02)	8:38 (1:03)
Glycated hemoglobin, %	5.4 (0.8)	5.5 (0.8)	5.3 (0.8)

Entries are mean (standard deviation) for continuous variables and number of observations and percent values for categorical variables.

The periodontal assessments were conducted using probing of periodontal pockets with a manual periodontal probe according to a half-mouth protocol (see **Supplement**). Mean probing pocket depth (PPD), proportion of sites with PPD ≥4 mm (pPPD4), proportion of sites with PPD ≥6 mm (pPPD6), mean clinical attachment level (CAL), proportion of interdental (mesiobuccal or distobuccal) CAL ≥2 mm (pINTCAL2), proportion of sites bleeding on probing (pBOP) served as outcome variables. Serum cortisol was sampled between 7 a.m. and 1 p.m. and stored at -80°C until analyses. The laboratory measurements were performed using a chemiluminescence immunoassay on the ADVIA CENTAUR XP (Siemens Healthcare Diagnostics, Eschborn, Germany) according to the manufacturer’s recommendations. The internal quality control was performed daily and showed coefficients of variation of 9.1% or 5.7% and 7.5% or 6.9% at the low (139 nmol/l) and high (1095 nmol/l) concentration in SHIP-TREND-0 or SHIP-START-2, respectively. We selected confounders using the modified disjunctive cause criterion (13). Direct causes of the exposure or outcome, excluding instrumental variables, were assumed to represent a sufficient confounder set. Accordingly, propensity scores were estimates using the following covariates: Age, sex, smoking status (former, never, current), alcohol consumption (grams of ethanol per day), waist circumference, fasting time, time of blood sampling, and glycated hemoglobin (14, 15). We further adjusted the models for baseline values of the outcomes to minimized the risk of reverse causation (16, 17).

### Statistical analysis

The associations of baseline cortisol with follow-up levels of PPD and CAL were examined using generalized linear models with gamma distribution and log link. The associations of baseline cortisol with follow-up pPPD4, pPPD6, pINTCAL2 and pBOP were modeled using fractional logit regressions. After confirming that linearity was met by testing cubic spline transformations, coefficients from these models were scaled per one standard deviation (1-SD). Effect estimates of gamma and fractional logit models were presented as 100x(e^β^-1) (i.e., relative change in outcome mean by adding 1-SD cortisol), with corresponding 95% confidence intervals (CIs) and P-values. We estimated a generalized propensity score (i.e., conditional density of cortisol, given covariates) using a convex Super Learner and adjusted for confounding using propensity score weighting (18). Super learning has been shown to provide optimal bias-variance tradeoff by weighting a combination of several prediction algorithms (19). We assessed balance using weighted correlations between each covariate and the continuous exposure, where correlations <0.1 indicate balance (20). To investigate whether selection bias due to attrition could be impacting on the complete case analysis, multiple imputation using chained equations was carried out to generate 20 imputed datasets. Effect estimates were pooled after propensity score weighting each dataset (21). Analyses were performed using the cobalt, MatchThem, mice, rms, SuperLearner, survey, stats, and WeightIt packages in R 4.1.3. The reporting was based on recommendations by STROBE (STrengthening the Reporting of OBservational studies in Epidemiology).

### Mendelian randomization study

MR is an application of instrumental variable analysis, which originated in econometrics, in the field of genetic epidemiology (22). It leverages the naturally occurring quasi-randomization of genotypes in the population, which largely reduces the risk of bias from environmental confounding and reverse causation. MR uses genetic variants as instruments for potentially modifiable risk factors. An instrument is a variable that affects the variable only through the exposure. A valid instrument satisfies three assumption (22): it should be strongly associated with the outcome (relevance assumption), does not share common causes with the outcome (exchangeability assumption), and influence the outcome only *via* its effect on the exposure (exclusion restriction). The relevance assumption can be tested by providing measures of instrument strength (23). The exchangeability and exclusion restriction assumptions can be violated by horizontal pleiotropy. Horizontal pleiotropy can occur when the genetic instrument is associated with confounders of the exposure-outcome association, is associated with risk factors of the outcome (i.e., correlated horizontal pleiotropy (24)), or if the instrument is directly associated with the outcome (25). Violations of the no horizontal pleiotropy assumption can be explored using sensitivity analysis (outlined below) (22, 25, 26). MR can be implemented by using genetic variants from specific genomic regions or across the whole genome (22, 27). Genome-wide association studies (GWAS) are used to identify genetic variants for the exposure phenotype. In two-sample MR, variant-exposure and variant-outcome associations originate from separate GWAS derived from the same superpopulation (22). In *cis*-MR, variants in single regions in proximity to genes of interest are selected (27). In polygenic MR, variants in each region are clumped for independence and only the variant with the smallest P-values are selected. Variants from different regions are combined to generate a set of polygenic instruments, thus increasing the statistical power to detect an association.

### Genetic instruments for cortisol and genetic association data for periodontitis

For primary analysis, instruments for cortisol were selected from the SERPINA1 and SERPINA6 loci on chromosome 14. The single nucleotide polymorphisms (SNPs) rs11621961, rs12589136, rs2749527 were associated with morning plasma cortisol at a P-value <5x10-8 in the CORtisol NETwork GWAS (28) meta-analysis containing 12,597 participants from 11 studies of European ancestry (**Tables 2**, **3**). Details on the participating studies are provided in **Supplementary Table 1**. GWAS analysis was adjusted for age, sex, and genetic principal components. SERPINA1 encodes α1-antitrypsin that inhibits cleavage of the reactive center loop that releases cortisol from cortisol-binding globulin and SERPINA6 encodes corticosteroid binding globulin (28). We additionally adopted a liberal selection strategy (P-value <5x10^-5^, linkage disequilibrium r² <0.2) to strengthen the instrument (29) and identified 35 instrumental SNPs (**Supplement Table 2**).

**Table 2:** Genome-wide association studies used for Mendelian randomization study.

Phenotype	Sample Size	Cases	Controls	Reference
Morning plasma cortisol	12597	–	–	(28)
Periodontitis	45563	17353	28210	(30)

**Table 3:** Summary of single nucleotide polymorphisms used to instrument plasma cortisol levels used in the primary analysis.

SNP	CHR	GENE	EA	OA	EAF	BETA	SE	P-value	F statistic
rs11621961	14	SERPINA6	T	C	0.357	-0.077	0.014	3.970e-08	30.2
rs12589136	14	SERPINA6	T	G	0.217	0.103	0.015	3.320e-12	48.6
rs2749527	14	SERPINA1	T	C	0.489	-0.081	0.012	5.210e-11	43.2

EA, effect allele; OA, other allele; EAF, effect allele frequency; SE, standard error.

GWAS summary statistics from a GWAS meta-analysis of 10 studies contributing of 17,353 periodontitis cases and 28,210 controls of European ancestry were obtained from the GeneLifestyle Interactions in Dental Endpoints consortium (GLIDE) (30) (**Table 2**, **Supplement Table 1**). Periodontitis cases were classified by either the Centers for Disease and Control and Prevention/American Academy of Periodontology (CDC/AAP) or the Community Periodontal Index (CPI) case definition.

### Statistical power to detect effect sizes in primary and secondary analyses

We calculated *a priori* statistical power according to Deng et al. (31). For the primary inverse-variance weighted (IVW) analysis, we had ≥80% power (α=5%) to detect an odd ratio (OR) of 1.30 for periodontitis per 1-SD increment in cortisol. For the secondary IVW analysis using the liberal instrument selection threshold, we had ≥80% power (α=5%) to detect an OR of 1.19 for periodontitis per 1-SD increment in cortisol.

### Statistical analysis

For the primary analysis using variants in the SERPINA1 and SERPINA6 loci, we computed Wald ratios for each SNP by dividing the effect estimate for the SNP-periodontitis association by the coefficient of the SNP-cortisol association (23). The correlation between SNPs was estimated using the 1000 Genomes Phase 3 reference panel. Wald ratios were pooled fixed-effects IVW for correlated instruments (27). The OR per 1-SD increment of cortisol was reported. IVW was repeated on the larger set of 35 SNPs selected using a liberal instrument selection criterion. To ensure that results from primary and secondary IVW analysis were not biased by horizontal pleiotropy, we used Cochran’s Q and I² to test heterogeneity of estimates, performed MR Egger’s intercept test for directional pleiotropy, and leave-one-out analysis to assess whether the IVW estimate was driven by a single SNP (26). As cortisol has obesogenic and hyperglycemic effects, and association of instrument SNPs with these traits might introduce correlated horizontal pleiotropy, we searched PhenoScanner to examine whether the instrumental SNPs had been found to be associated (P-value <5x10^-5^) with body mass index, glucose, glycated hemoglobin or insulin (25). For the secondary analysis, we applied additional pleiotropy-robust models: Generalized Summary data-based Mendelian randomization (GSMR) (32), robust adjusted profile score (RAPS) (33), Causal Analysis Using Summary Effect Estimates (CAUSE) (24). By leveraging 10,000 SNP instruments, CAUSE improves statistical power and lowers the risk of weak instrument bias. Each method makes different assumptions and therefore a consistent effect across multiple methods strengthens MR evidence. We performed MR analyses in R using the cause, gsmr, MendelianRandomization, mr.raps, and TwoSampleMR packages. The analysis follows STROBE-MR (Strengthening the Reporting of Observational Studies in Epidemiology using Mendelian Randomization). As SNP-level data shared from GWAS are deidentified, no additional ethical approval is required.

## Results

### Observational study

**Table 1** provides baseline descriptive statistics of confounders in the analytic sample of 3,388 individuals. The mean age was 50.8 years, 52% were female, and 40.2% and 38.6% were current and former smokers, respectively. The mean average alcohol consumption was 9.5 grams of pure alcohol per day, mean waist circumference was 89.6 cm, average fasting time was 8:14 h:min, average fasting time was 9:18 h:min, and average glycated hemoglobin was 5.4%. Confounders were well balanced after propensity score weighting (i.e., weighted correlations coefficients <0.1) (**Supplement Figure 1**). In the complete case sample, propensity score weighting revealed associations of baseline cortisol with follow-up CAL (100(e^β^-1) per 1-SD = 1.64, 95% CI: 0.47;2.81, P-value=0.006), pINTCAL2 (100(e^β^-1) per 1-SD = 6.60, 95% CI: 2.38;10.63, P-value=0.002) and pBOP (100(e^β^-1) per 1-SD = 4.50, 95% CI: 0.16;9.01, P-value=0.042) (**Table 4**). The positive association between baseline cortisol and follow-up pBOP persisted after multiple imputation, although the effect size was attenuated (**Supplement Table 3**). The complete case and multiple imputation analyses showed no association of baseline cortisol with follow-up PPD, pPPD4, or pPPD6 (**Table 4**, **Supplement Table 3**).

**Table 4:** Confounder-adjusted associations between baseline serum cortisol and follow-up periodontal measurements in complete case analysis of the Study of Health in Pomerania.

	100(e^β^-1)	95% confidence interval		*P*-value
Mean pocket probing depth	0.29	-0.35	0.93	0.3711
Proportion of sites with pocket probing depth ≥4 mm	3.77	-1.38	8.66	0.1481
Proportion of sites with pocket probing depth ≥6 mm	5.45	-6.60	16.14	0.3601
Mean clinical attachment level	1.64	0.47	2.81	0.0062
Proportion of interdental clinical attachment level >2mm	6.60	2.38	10.63	0.0024
Proportion of sites bleeding on probing	4.50	0.16	9.01	0.0415

Gamma regression: mean pocket probing depth, mean clinical attachment level. Fractional logit model: proportion of sites with mean pocket probing depth ≥4 mm, proportion of sites bleeding on probe. Confounder-adjustment using propensity score weighting. Adjusted for baseline value of outcome variable, age, sex, smoking, alcohol consumption, waist circumference, time of blood sampling, fasting time, and glycated hemoglobin.

### Mendelian randomization study

The primary MR analysis using three SNPs had a minimum F-statistics of 30.2 and explained 0.5% of the variance in plasma cortisol (**Table 3**). Using the three primary SNPs, the IVW OR for periodontitis was 1.01 (95% CI: 0.57;1.81, P-value=0.971) per 1-SD increment of cortisol. There was no heterogeneity in the primary IVW analysis (**Supplement Table 4**). The MR Egger intercept was centered around zero and suggested no unbalanced pleiotropy (**Supplement Table 4**). The leave-one-out analysis revealed no outlier SNP (**Supplement Table 5**).

**Figure 1:**
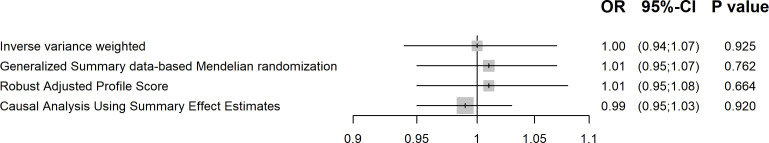
Inverse variance and pleiotropy-robust models for secondary analysis. OR, odds ratio per standard deviation increment in morning plasma cortisol. CI: confidence interval (Causal Analysis Using Summary Effect Estimates: Bayesian 95% credible interval).

Using the 35 SNPs identified using a liberal instrument selection criterion, the secondary analysis had a minimum F statistic of 16.7 and explained 1.6% of the variance in morning plasma cortisol (**Supplement Table 2**). The IVW OR was 1.00 (95% CI: 0.94;1.07, P-value=0.925) (**Figure 1**). Heterogeneity analysis, MR Egger’s test and leave-one out analysis suggested no pleiotropy (**Supplement Tables 4**, **5**). Additional pleiotropy-robust analyses using GSMR, RAPS and CAUSE supported the IVW results (**Figure 1**). The PhenoScanner search revealed no reported associations with body mass index, glucose, glycated hemoglobin, or insulin in primary and secondary analyses, providing reassurance that estimates were not subject to correlated horizontal pleiotropy.

## Discussion

The present observational cohort and MR studies found diverging evidence for an effect of circulating cortisol levels on periodontitis. The observational study examined serum cortisol and suggested modestly sized positive associations with follow-up periodontitis markers. In contrast, the MR study investigated the effect of genetically instrumented long-term plasma cortisol on periodontitis risk and did not support an effect.

In the observational analyses, cortisol was associated with CAL and BOP. CAL reflects cumulative periodontal disease history, whereas BOP indicates recent periodontal inflammation. The prospective association between circulating cortisol and periodontal measures seen in the cohort analysis is in line with previous cross-sectional research that showed a positive association between cortisol in saliva and serum and periodontitis (2, 10). Additionally, case-control studies indicated that cortisol levels were higher in subjects with periodontal disease than in controls (2, 10, 11). For example, one study including 90 subjects found that serum cortisol was lower in non-smoking controls than in smoking and non-smoking periodontitis cases (34). A cross-sectional study of 467 older subjects found a positive association between serum cortisol and periodontitis severity, as shown by CAL (35). Yet, in cross-sectional and case-control studies it is unclear whether higher cortisol precedes periodontitis or is elevated because of changes in periodontal health. We controlled for baseline values of periodontal measures, which reduces the possibility that periodontitis-induced increase in cortisol could have introduced a reverse-causal association (17). The cohort study had potential limitations. First, we controlled the cohort analysis for several potential confounding factors at baseline. In doing so, we hoped to minimize potential bias associated with these confounding factors. However, we cannot exclude the possibility that unmeasured behavioral, psychological, or clinical common causes of cortisol and periodontitis, potentially changing as consequence of cortisol at baseline (i.e., time-varying confounding), biased the cohort analysis. Second, cortisol was measured once between 7 a.m. and 1 p.m. blood samples, although it has large diurnal variation which might have biased regression coefficients. We sought to minimize the influence of diurnal fluctuation by including blood sampling time as a covariate in propensity score weighting. Methodological work has shown that this is a feasible strategy and that associations are usually strengthened by adjusting for time of sampling (36). Future studies examining salivary cortisol should ideally obtain morning cortisol levels, the awakening response, and the slope in cortisol decline across the day. Because salivary cortisol is more proximal to the periodontium, it is expected to show stronger association with periodontal measures. Alternatively, cortisol in hair provides a cumulative long-term exposure (37). Such observational evaluation of cumulative cortisol exposure in hair would also parallel the long-term exposure that is assessed in MR. Third, the effect of error-prone periodontal outcome measurements induced by using a half mouth periodontal examination protocol could have attenuated gamma regression and fractional logit estimates towards the null given that the measurement error in the prospective design is expected to act non-differential and independent with respect to the exposure.

Although no previous study has examined the effect of genetically instrumented cortisol on periodontitis, previous MR studies have shown that the approach is generally suitable to triangulate observational estimates of cortisol exposure (38–40). The MR approach is less likely to be biased by observational confounding or reverse causation, for example, cortisol release as a consequence of periodontitis-induced local or systemic inflammation. Particularly for cortisol, a recent MR study using the same genetic instruments failed to demonstrate effects on type 2 diabetes and cardiovascular disease, outcomes with similar obesogenic and hyperglycemic pathways (38). Conversely, a MR study provided evidence to support an effect of cortisol on circulating interleukin 8 and macrophage migratory inhibitory factor (39). A wide-angle MR study using the same variants indicated that elevated cortisol may increase the risk of endometrial cancer (40). The MR study had potential limitations. First, the MR study has few instruments, which might have introduced weak instrument bias and low statistical power. Larger GWAS of cortisol could provide additional instruments for this trait and larger power for its effect on periodontitis. Recently, a larger GWAS of plasma morning cortisol was published (41). However, we did not retrieve SNP-cortisol associations from this GWAS because of partial participant overlap with the periodontitis GWAS, which would have biased the MR estimate towards the observational estimate (22). Second, the MR study was based on GWAS of morning cortisol. It is possible that evening cortisol, diurnal variation (i.e., cortisol slope) or salivary cortisol are more relevant to capture periodontal risk. Unfortunately, only GWAS of morning cortisol are available. Additionally, it is unknown whether SNP-cortisol associations vary with age. Thus, the present MR study could not assess whether cumulative cortisol or cortisol at critical life stages matter, and if there is any critical timing of exposure measurement. Instead, it tested the global null hypothesis of no effect for any individuals (i.e. there is no part of the liability trajectory that causes the outcome (42)). Finally, our MR study was based on GWAS with participants of European descent and is it unclear whether findings also apply to other populations.

In conclusion, we did not find robust evidence for an effect of cortisol on periodontitis. The bias sources of the observational and MR studies are expected to differ. While the cohort analysis may be subject to residual confounding (by environmental and clinical phenotypes) and measurement error, the MR study could be biased by horizontal pleiotropic pathways not detected through PhenoScanner or weak instruments and could be subject to low statistical power. Generally, aligned findings of observational and MR research would strengthen the conclusion that there is a causal effect (43, 44). The study casts some doubt on a cortisol-related pathway in periodontitis and it may stimulate further research regarding the involvement of glucocorticoids, more generally the HPA axis, in periodontal health.

## Data availability statement

The data analyzed in this study is subject to the following licenses/restrictions: Research proposals for using data from the Study of Health of Pomerania can be posted at http://ship.community-medicine.de. Requests to access these datasets should be directed to http://ship.community-medicine.de.

## Ethics statement

The studies involving human participants were reviewed and approved by Ethics Committee of the University of Greifswald. The patients/participants provided their written informed consent to participate in this study.

## Author contributions

S-EB, SR, NF, BH contributed to conception, design, data acquisition, performed the analysis, and drafted the manuscript. H-JG, MN, JK-K, TK and HV contributed to data acquisition and interpretation, and critically revised the manuscript. All authors contributed to the article and approved the submitted version.
