# Supplementary material for: Cortisol and periodontitis: Prospective observational and Mendelian randomization studies
Source: Front Endocrinol (Lausanne). 2023 Mar 15;14:1100985. doi: 10.3389/fendo.2023.1100985 (PMC10050732; doi:10.3389/fendo.2023.1100985)
Supplement: Supplementary file 1 [file DataSheet_1.docx]

# Cortisol and periodontitis: prospective observational and Mendelian randomization studies

Sebastian-Edgar Baumeister et al.

Supplement

## Periodontal measurements

Measurements were assessed with a manual periodontal probe (SHIP-START-2/3: PCP-11; SHIP-TREND: PCP15; Hu-Friedy, USA) at mesiobuccal, midbuccal, distobuccal and midpalatinal/midlingual tooth sites for all teeth except third molars, according to a half-mouth protocol (1). Probing pocket depth (PPD) equals the distance from the free gingival margin to the bottom of the periodontal pocket. The clinical attachment level (CAL) equals the distance from the cemento-enamel junction to the pocket base. CAL was not recorded when cemento-enamel junction determination was indistinct (e.g. wedge-shaped defects, fillings and crown margins). Measurements were rounded to the closest millimeter. All available site measurements were pooled over sites and teeth (56 at maximum; 4 sites at 14 teeth) and used for calculation of mean PPD and mean CAL, which reflect current and life-time accumulated periodontal disease severity, respectively. Bleeding on probing was recorded at four identical sites on the first incisor, the canine, and the first molar in each probed quadrant. If teeth were missing, the next distally located tooth was assessed. The proportion of sites bleeding on probing was determined.

Dental examinations were conducted by calibrated and licensed dentists. Biannual calibration exercises were conducted on people not connected to the study. In SHIP-START, dental examinations were conducted by six examiners. Intra-rater correlations of 0.70–0.89 per examiner and an inter-rater correlation of 0.90 for CAL measurements were achieved. For PPD measurements, intra-rater correlations ranged between 0.43–0.82 per examiner and pairwise inter-rater correlations ranged between 0.41 and 0.78. In SHIP-Trend, dental examinations were conducted by five examiners. Intra-rater correlations for CAL measurements ranged between 0.67 and 0.89 and inter-rater correlation was 0.70. For PPD measurements, the examiners yielded intra-rater correlations between 0.68 and 0.88 and an inter-rater correlation of 0.72.

Supplement Figure 1 Correlation-based covariate balancing diagnostics


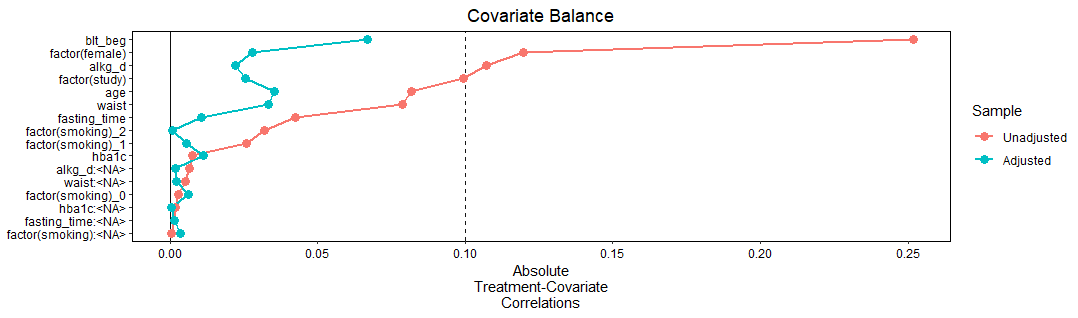


Supplement Table 1 Phenotypic descriptive statistics of studies included in the cortisol and periodontitis genome-wide association studies

| GWAS / Study | Phenotype | | | | |
| --- | --- | --- | --- | --- | --- |
| Bolton et al. (2) | Morning plasma cortisol | | | | |
|  | N | Age mean (sd) | Female % | Plasma cortisol mean (sd) | Sampling hours |
| ORCADES | 886 | 53.5 (15.7) | 54.6 | 765 (315) | 0830-1030 |
| CROATIA-Korcula | 898 | 56.2 (13.9) | 63.8 | 698 (207) | 0800-0900 |
| CROATIA-Split | 496 | 45.0 (14.7) | 57.1 | 979 (404) | 0730-0900 |
| CROATIA-Vis | 892 | 56.4 (15.5) | 56.5 | 622 (230) | 0730-0900 |
| Rotterdam Study | 2945 | 71.9 (7.0) | 56.4 | 305 (94) | 0800-1100 |
| HBCS1934-44 | 451 | 60.61 (2.80) | 63.9 | 393 (120) | 0750-1055 |
| NFBC1966 | 1,192 | 31(0) | 100 | 380 (160) | 0800-1100 |
| ALSPAC | 1,567 | 15.43 (0.26) | 49.7 | 486 (174) | 0800-1057 |
| InChianti | 1,210 | 68.3 (15.6) | 55.5 | 375 (135) | Before 0900 |
| PIVUS | 919 | 70.2 (0.17) | 49,8 | 386 (125) | 0800-1000 |
| PREVEND | 1151 | 49.4 (13.0) | 49.4 | 442 (201) | 0800-1100 |
| Shungin et al. (3) | Periodontitis | | | | |
|  | N | Age mean (sd) | Female % | Periodontitis % | |
| GLACIER | 4,041 | 51.9 (9.0) | 63.1 | 36.3 | |
| MDC | 4,684 | 59.3 (9.4) | 63.7 | 64.6 | |
| TwinGene | 2,849 | 63.6 (8.0) | 53.6 | 53.4 | |
| ARIC | 4,504 | 62.7 (5.6) | 52 | 59.5 | |
| COHRA | 772 | 33.9 (9.0) | 62.7 | 20.8 | |
| SHIP | 3,264 | 45.8 (14.9) | 51.1 | 55.5 | |
| SHIP-TREND | 819 | 49.2 (13.2) | 55.1 | 51.8 | |
| WGHS | 22,888 | 54.7 (7.1) | 100 | 24.7 | |
| NHS | 7,404 | 65.5 (6.5) | 100 | 15.7 | |
| HPFS | 5,912 | 55.6 (8.5) | 0 | 26.1 | |

ALSPAC, Avon Longitudinal Study of Parents and Children. ARIC, Atherosclerosis Risk in Communities. COHRA, Center for Oral Health Research in Applachia. CROATIA, 100001 Dalmations The Croatian Biobank. GLACIER, Gene x Lifestyle interactions And Complex traits Involved in Elevated disease Risk. HBCS1934-44, Helsinki Birth Cohort 1934-44 Study. HPFS, Health Professionals Follow-up Study. InChianti, Invecchiare in Chianti, aging in Chianti. MDC, Malmö Diet and Cancer Study. NFBC1966, Northern Finland Birth Cohort 1966. NHS, Nurses' Health Study. ORCADES, Orkney Complex Disease. PIVUS, Prospective Investigation of the Vasculature in Uppsala Seniors. PREVEND, Prevention of Renal and Vascular End-stage Disease. Rotterdam Study, Rotterdam Study. SHIP, Study of Health in Pomerania. SHIP-Trend, Study of Health in Pomerania Trend. TwinGene, TwinGene Study. WGHS, Women's Genome Health Study.

Supplement Table 2 Summary of single nucleotide polymorphisms used to instrument plasma cortisol levels used in the secondary analysis

| SNP | CHR | GENE | EA | OA | EAF | BETA | SE | P-value | F statistic |
| --- | --- | --- | --- | --- | --- | --- | --- | --- | --- |
| rs10452224 | 4 | NR3C2 | G | A | 0.443 | -0.052 | 0.013 | 3.550e-05 | 17.1 |
| rs10495661 | 2 | RAD51AP2 | C | G | 0.244 | -0.061 | 0.015 | 2.740e-05 | 17.6 |
| rs1075533 | 11 | DYNC2H1 | A | G | 0.037 | -0.172 | 0.035 | 7.740e-07 | 24.5 |
| rs11078021 | 17 | DNAH9 | T | C | 0.271 | -0.063 | 0.015 | 3.730e-05 | 17.0 |
| rs11568283 | 12 | CD9 | G | A | 0.030 | 0.237 | 0.057 | 3.080e-05 | 17.4 |
| rs11621961 | 14 | SERPINA6 | T | C | 0.357 | -0.077 | 0.014 | 3.970e-08 | 30.2 |
| rs11717324 | 3 | MIR548A3 | A | G | 0.271 | 0.059 | 0.014 | 3.920e-05 | 16.9 |
| rs11934518 | 4 | MGC45800 | C | T | 0.109 | -0.082 | 0.020 | 3.980e-05 | 16.9 |
| rs12589136 | 14 | SERPINA6 | T | G | 0.217 | 0.103 | 0.015 | 3.320e-12 | 48.6 |
| rs1340395 | 1 | OLFM3 | T | C | 0.926 | -0.126 | 0.026 | 1.090e-06 | 23.8 |
| rs1473492 | 15 | RGMA | T | C | 0.971 | 0.207 | 0.048 | 1.450e-05 | 18.8 |
| rs1591208 | 6 | OLIG3 | T | C | 0.250 | 0.061 | 0.015 | 4.400e-05 | 16.7 |
| rs17029942 | 3 | CRBN | A | G | 0.026 | 0.653 | 0.128 | 3.100e-07 | 26.2 |
| rs17311146 | 8 | RSPO2 | T | C | 0.168 | 0.068 | 0.017 | 4.380e-05 | 16.7 |
| rs2061471 | 7 | BZW2 | T | C | 0.327 | 0.057 | 0.014 | 3.290e-05 | 17.3 |
| rs2144835 | 14 | SERPINA6 | T | C | 0.709 | 0.076 | 0.015 | 3.380e-07 | 26.1 |
| rs2354973 | 7 | ZC3HAV1L | C | A | 0.504 | 0.065 | 0.015 | 1.030e-05 | 19.5 |
| rs2427453 | 20 | GID8 | T | C | 0.485 | -0.056 | 0.013 | 2.780e-05 | 17.6 |
| rs2822584 | 21 | HSPA13 | A | T | 0.061 | -0.116 | 0.027 | 1.490e-05 | 18.8 |
| rs40718 | 5 | SEMA5A | A | G | 0.508 | -0.053 | 0.013 | 2.180e-05 | 18.1 |
| rs4147799 | 1 | ABCA4 | G | A | 0.023 | 0.537 | 0.125 | 1.760e-05 | 18.5 |
| rs4400057 | 4 | LOC255130 | A | G | 0.912 | -0.319 | 0.065 | 9.460e-07 | 24.1 |
| rs6017731 | 20 | SLC12A5 | C | T | 0.947 | 0.127 | 0.029 | 1.550e-05 | 18.7 |
| rs6070847 | 20 | PHACTR3 | G | A | 0.161 | 0.086 | 0.019 | 6.260e-06 | 20.4 |
| rs6122963 | 20 | PARD6B | G | C | 0.072 | -0.105 | 0.024 | 1.680e-05 | 18.5 |
| rs6830 | 14 | DNAL1 | A | G | 0.320 | -0.063 | 0.013 | 1.940e-06 | 22.7 |
| rs6923385 | 6 | CD109 | G | C | 0.024 | 0.428 | 0.105 | 4.540e-05 | 16.7 |
| rs7090949 | 10 | SORCS3 | C | T | 0.256 | 0.064 | 0.014 | 7.170e-06 | 20.2 |
| rs7116606 | 11 | KCNJ1 | C | T | 0.710 | 0.063 | 0.015 | 2.080e-05 | 18.1 |
| rs7161231 | 14 | SERPINA6 | T | C | 0.115 | 0.084 | 0.020 | 4.060e-05 | 16.9 |
| rs7192943 | 16 | LOC100506172 | G | C | 0.225 | 0.075 | 0.018 | 3.600e-05 | 17.1 |
| rs7217509 | 17 | CPSF4L | G | C | 0.206 | 0.102 | 0.024 | 2.030e-05 | 18.2 |
| rs7771144 | 6 | EYA4 | T | A | 0.147 | -0.079 | 0.019 | 2.140e-05 | 18.1 |
| rs941598 | 14 | SERPINA6 | T | C | 0.406 | -0.059 | 0.013 | 3.260e-06 | 21.7 |
| rs9630649 | 16 | ATP2C2 | C | T | 0.217 | -0.066 | 0.015 | 1.890e-05 | 18.3 |

EA, effect allele. OA, other allele. EAF, effect allele frequency. SE, standard error

Supplement Table 3 Confounder-adjusted associations between baseline cortisol and follow-up periodontal measurements using multiple imputation in the Study of Health in Pomerania

|  | 100(e^β^-1) | 95% confidence interval | | *P*-value |
| --- | --- | --- | --- | --- |
| Mean pocket probing depth | 0.27 | -0.35 | 0.88 | 0.3954 |
| Proportion of sites with pocket probing depth ≥4 mm | 3.55 | -1.42 | 8.28 | 0.1618 |
| Proportion of sites with pocket probing depth ≥6 mm | 3.56 | -6.78 | 12.90 | 0.4865 |
| Mean clinical attachment level | 1.22 | -0.05 | 2.48 | 0.0637 |
| Proportion of interdental clinical attachment level >2mm | 3.98 | -0.71 | 8.46 | 0.0983 |
| Proportion of sites bleeding on probing | 3.56 | 0.24 | 6.98 | 0.0355 |

Gamma regression: mean pocket probing depth, mean clinical attachment level. Fractional logit model: proportion of sites with mean pocket probing depth ≥4 mm, proportion of sites bleeding on probe. Confounder-adjustment using propensity score weighting. Adjusted for baseline value, age, sex, smoking, alcohol consumption, waist circumference, time of blood sampling, fasting time, and glycated hemoglobin

Supplementary Table 4 Heterogeneity of Wald ratios and MR-Egger test for directional pleiotropy

| Exposure | Heterogeneity | | |  |
| --- | --- | --- | --- | --- |
|  | Q | Degrees of Freedom | P | I² |
| Primary analysis | 4.46 | 2 | 1.074e-01 | 0.366 |
| Secondary analysis | 39.5 | 34 | 2.378e-01 | 0.117 |
|  | MR-Egger test for directional pleiotropy | | |  |
|  | Intercept | Standard error | P |  |
| Primary analysis | -1.528e-01 | 0.077 | 2.985e-01 |  |
| Secondary analysis | -3.213e-03 | 0.006 | 5.818e-01 |  |

Supplementary Table 5 Inverse variance weighted estimates in leave-one-out analysis in primary and secondary analysis

| SNP excluded | SNP | OR | (95% CI) | P value |
| --- | --- | --- | --- | --- |
| Primary analysis | rs11621961 | 1.02 | (0.61;1.72) | 0.9259 |
|  | rs12589136 | 1.01 | (0.75;1.89) | 0.710 |
|  | rs2749527 | 1.10 | (0.72;1.68) | 0.6710 |
| Secondary analysis | rs10452224 | 1.02 | (0.96;1.09) | 0.4989 |
|  | rs10495661 | 1.02 | (0.96;1.09) | 0.4759 |
|  | rs1075533 | 1.02 | (0.96;1.09) | 0.4917 |
|  | rs11078021 | 1.02 | (0.96;1.09) | 0.5145 |
|  | rs11568283 | 1.01 | (0.95;1.08) | 0.6561 |
|  | rs11621961 | 1.02 | (0.96;1.09) | 0.4857 |
|  | rs11717324 | 1.02 | (0.95;1.09) | 0.5965 |
|  | rs11934518 | 1.02 | (0.95;1.08) | 0.6146 |
|  | rs12589136 | 1.01 | (0.95;1.08) | 0.7055 |
|  | rs1340395 | 1.02 | (0.95;1.09) | 0.5716 |
|  | rs1473492 | 1.02 | (0.96;1.09) | 0.5101 |
|  | rs1591208 | 1.01 | (0.95;1.08) | 0.6637 |
|  | rs17029942 | 1.03 | (0.96;1.11) | 0.4443 |
|  | rs17311146 | 1.02 | (0.96;1.09) | 0.4710 |
|  | rs2061471 | 1.02 | (0.96;1.09) | 0.4962 |
|  | rs2144835 | 1.02 | (0.96;1.09) | 0.5264 |
|  | rs2354973 | 1.02 | (0.96;1.09) | 0.4794 |
|  | rs2427453 | 1.02 | (0.96;1.09) | 0.4838 |
|  | rs2822584 | 1.02 | (0.95;1.08) | 0.6196 |
|  | rs40718 | 1.02 | (0.96;1.09) | 0.5244 |
|  | rs4147799 | 1.01 | (0.95;1.08) | 0.7162 |
|  | rs4400057 | 1.01 | (0.94;1.09) | 0.6910 |
|  | rs6017731 | 1.03 | (0.97;1.09) | 0.3701 |
|  | rs6070847 | 1.02 | (0.95;1.09) | 0.5925 |
|  | rs6122963 | 1.02 | (0.96;1.09) | 0.5471 |
|  | rs6830 | 1.02 | (0.96;1.09) | 0.4695 |
|  | rs6923385 | 1.02 | (0.95;1.09) | 0.6098 |
|  | rs7090949 | 1.02 | (0.96;1.09) | 0.5453 |
|  | rs7116606 | 1.02 | (0.95;1.09) | 0.6031 |
|  | rs7161231 | 1.02 | (0.96;1.09) | 0.5173 |
|  | rs7192943 | 1.01 | (0.95;1.08) | 0.6541 |
|  | rs7217509 | 1.02 | (0.96;1.09) | 0.5178 |
|  | rs7771144 | 1.01 | (0.95;1.07) | 0.7340 |
|  | rs941598 | 1.02 | (0.95;1.09) | 0.5638 |
|  | rs9630649 | 1.02 | (0.95;1.08) | 0.6144 |

References

1. Holtfreter B, Stubbe B, Gläser S, Trabandt J, Völzke H, Ewert R, et al. Periodontitis Is Related to Exercise Capacity: Two Cross-sectional Studies. *Journal of dental research* (2021) **100**:824–32. doi:10.1177/0022034521995428.

2. Bolton JL, Hayward C, Direk N, Lewis JG, Hammond GL, Hill LA, et al. Genome wide association identifies common variants at the SERPINA6/SERPINA1 locus influencing plasma cortisol and corticosteroid binding globulin. *PLoS genetics* (2014) **10**:e1004474. doi:10.1371/journal.pgen.1004474.

3. Shungin D, Haworth S, Divaris K, Agler CS, Kamatani Y, Keun Lee M, et al. Genome-wide analysis of dental caries and periodontitis combining clinical and self-reported data. *Nature communications* (2019) **10**:2773. doi:10.1038/s41467-019-10630-1.
